# Anti-Influenza Virus (H5N1) Activity Screening on the Phloroglucinols from Rhizomes of *Dryopteris crassirhizoma*

**DOI:** 10.3390/molecules22030431

**Published:** 2017-03-08

**Authors:** Juan Wang, Yan-Tao Yan, Shen-Zhen Fu, Bing Peng, Lin-Lin Bao, Yan-Ling Zhang, Jing-Hong Hu, Zu-Ping Zeng, Dong-Hao Geng, Zeng-Ping Gao

**Affiliations:** 1 School of Chinese Materia Medica, Beijing University of Chinese Medicine, Beijing 100102, China; juan_wangli@163.com (J.W.); yanyt296@163.com (Y.-T.Y.); fushenzhen1988@163.com (S.-Z.F.); collean_zhang@163.com (Y.-L.Z.); 2 Beijing Institute of Traditional Chinese Medicine, Beijing Hospital of Traditional Chinese Medicine Affiliated to Capital Medical University, Beijing 100000, China; pengbing126@126.com (B.P.); zzp600@sohu.com (Z.-P.Z.); 3 Institute of Laboratory Animal Sciences, Chinese Academy of Medical Sciences & Peking Union Medical College (CAMS&PUMC), Beijing 100730, China; bllmsl@aliyun.com; 4 School of Basic Medical Science, Beijing University of Chinese Medicine, Beijing 100029, China; hujhbj@163.com; 5 College of Pharmacy, China Pharmaceutical University, Nanjing 211198, China; gengdonghao@163.com

**Keywords:** *Dryopteris crassirhizoma*, phloroglucinols, influenza virus H5N1, neuraminidase, dryocrassin ABBA

## Abstract

For screening the active phloroglucinols on influenza virus (H5N1) from *Dryopteris crassirhizoma* NaKai, a database was established including twenty-three phloroglucinols that had been isolated from *Dryopteris crassirhizoma*. Their inhibitory effect on the neuraminidase (NA) of influenza virus H5N1 was screened by molecular docking. As a result, three candidates were selected. The rhizomes of *D. crassirhizoma* were subjected to isolation and purification processes to obtain the inhibitor candidates. Thirteen phloroglucinols were obtained, including three selected candidates and two new phloroglucinols. The five phloroglucinols were investigated for their inhibitory activity on NA in vitro. The results showed that dryocrassin ABBA and filixic acid ABA exhibited inhibitory effects on NA with IC_50_ as 18.59 ± 4.53 and 29.57 ± 2.48 μM, respectively, and the other three phloroglucinols showed moderate inhibitory activity. Moreover, the anti-influenza virus (H5N1) activity and cytotoxicity of dryocrassin ABBA and filixic acid ABA were tested on Madin-Darby canine kidney (MDCK) cells with the cell counting kit-8 (CCK8) method. The results confirmed that dryocrassin ABBA exhibited an inhibitory activity with low cytotoxicity (TC_50_ > 400 μM) against influenza virus (H5N1) which will have to be investigated in further detail. In conclusion, phloroglucinols from *D. crassirhizoma* were shown to have anti-influenza virus activity, and especially dryocrassin ABBA, one of the phloroglucinols, may have the potential to control influenza virus (H5N1) infection.

## 1. Introduction

Highly pathogenic avian influenza virus (HPAIV), subtype H5N1, is of great concern for infecting and causing an inordinate amount of lethal disease in humans that could evolve into a pandemic risk [1]. Human H5N1 influenza infection was first recognized in 1997 when this virus infected 18 people in Hong Kong, causing six deaths [2,3]. According to the World Health Organization, a total of 665 people were infected with the H5N1 virus from 2003 to 2014, resulting in 392 deaths, with a mortality rate of 58.9%. Vaccine is an effective measure for preventing infection, but virus mutations are abrupt and rapid, so vaccines would be developed too late to be used for prevention during the more severe phases of recent pandemics. Amantadine, rimantadine, oseltamivir, and zanamivir have been approved by the United States Food and Drug Administration for the treatment of pandemic influenza. Studies have demonstrated that many of the avian influenza H5N1 viruses were resistant to both amantadine and rimantadine [4]. In addition, an H5N1 virus that was resistant to the drug oseltamivir has been isolated by Le et al. [5]. Therefore, much attention has been paid to Chinese herbs as potential sources for the discovery of new anti-influenza compounds with high efficacy and low toxicity.

*D. crassirhizoma* (DC) is widely distributed in the northeast of China and rhizome has been traditionally used for treating various inflammatory [6,7,8,9] and infectious diseases [10,11]. Many studies demonstrated that phloroglucinols from DC had a wide range of pharmacological effects, such as antibacterial [12], anti-influenza virus [13], anti-tumor promoting [14,15], anti-reverse transcriptase [16], and antioxidant activities [17]. Furthermore, flavaspidic acid AB, one of the phloroglucinols from this plant, was able to inhibit replication of the porcine reproductive and respiratory syndrome virus (PRRSV) [18]. Our pilot study suggested that the extract fraction of this plant that was rich in phloroglucinols could reduce mortality and decrease lung index (dry lung-to-body weight ratio) of mice inoculated with avian influenza virus (H5N1). Dryocrassin ABBA was isolated from this fraction, which could protect mice infected with avian influenza virus via inhibiting inflammation and reducing viral loads [19]. Therefore, anti-influenza virus activity screening on phloroglucinols was carried out as a part of our program exploring the most active phloroglucinol from DC. In this report, virtual screening with molecular docking, isolation, structural characterization, and the activity on neuraminidase (NA) and influenza virus in vitro are described.

## 2. Results and Discussion

### 2.1. Molecular Docking

**Figure 1:**
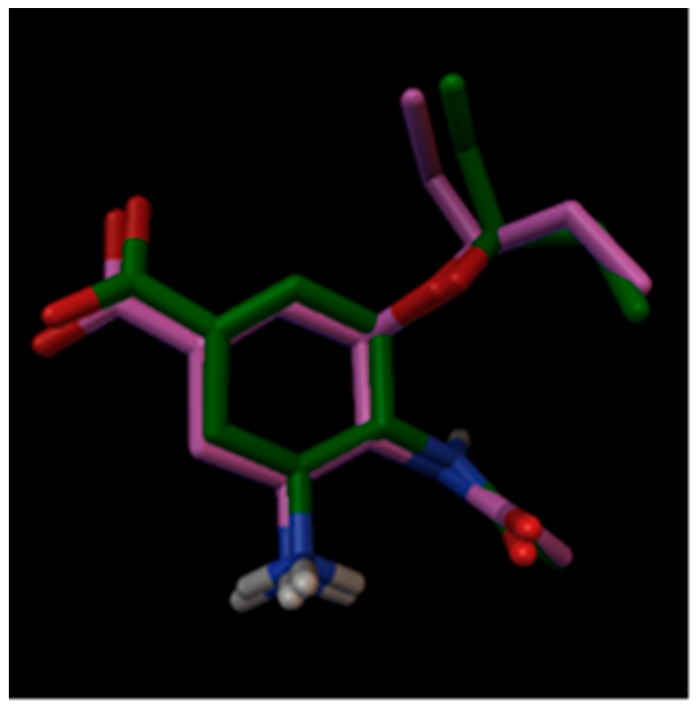
Co-crystallized (green) and redocked (pink) oseltamivir. The reliability of molecular docking was validated using the known X-ray structure of neuraminidase (NA) complexed with oseltamivir. The result demonstrated that the low root mean-square deviation (RMSD) was 1.12 Å between the docked and the crystal conformation of oseltamivir, which indicated the high reliability of AutoDock Vina.

Twenty-three phloroglucinols that had been isolated from DC were selected from the databases of CNKI and Web of Science (Table S1). To determine the probable binding conformations of the phloroglucinols with NA, Autodock Vina was used to dock them into the active site of NA (N1 Neuraminidase H274Y, PDB ID: 3CL0), isolated from H5N1-infected patients that have been reported to be oseltamivir-resistant [20]. The docking reliability was studied using the known X-ray structure of NA complexed with oseltamivir. As shown in Figure 1, the low root mean-square deviation (RMSD) was 1.12 Å between the docked and the crystal conformation of oseltamivir, which indicated the high reliability of AutoDock Vina in reproducing the experimentally observed binding mode for the NA inhibitor. The results exhibited that the minimum binding energy of the twenty-three phloroglucinols ranged from –6.3 to –8.3 kcal/mol. The lowest binding energy of the protein-ligand complex was considered as the best [21]. Hence, the binding conformations of twenty-three phloroglucinols with a docking score ≤−8.0 were chosen. Finally, ten phloroglucinols were selected with the lowest docking energies of the protein-ligand complex (Table 1).

**Figure 2:**
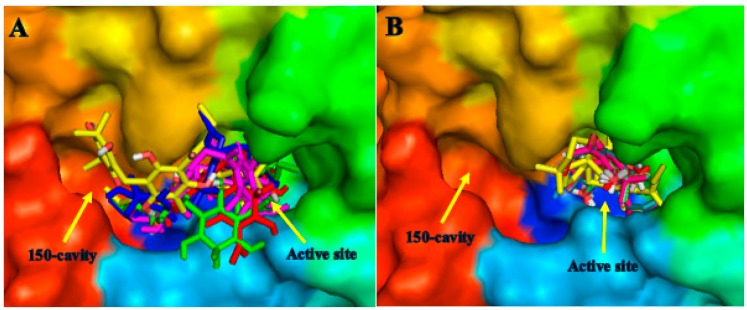
Binding modes of trimeric, tetrameric phloroglucinols (**A**) and monomeric, dimer phloroglucinols (**B**) in the active site and ‘150-cavity’ of NA.

**Figure 3:**
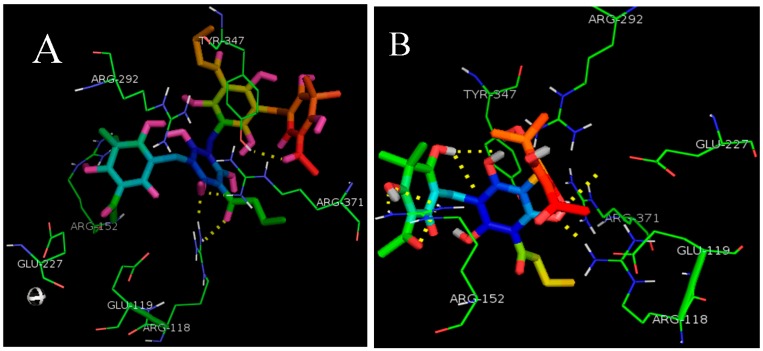

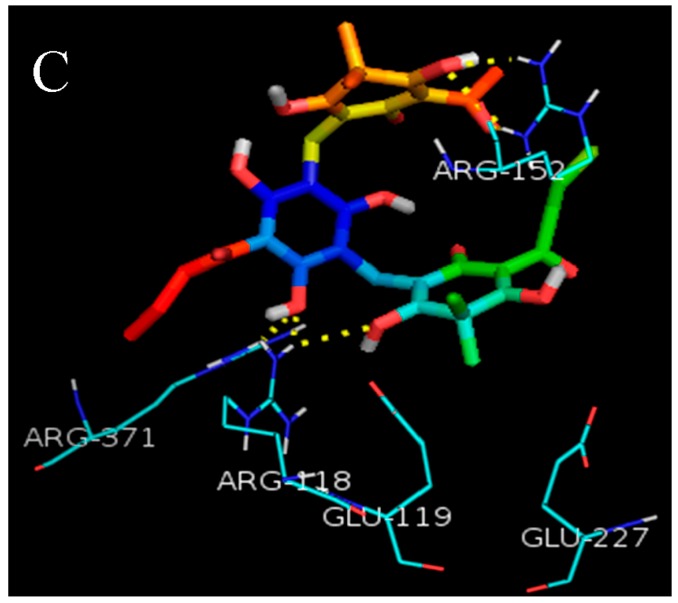
Binding modes of dryocrassin ABBA (**A**); filixic acid ABA (**B**) and filixic acid ABB (**C**) with key residues of NA.

It has been reported that there was a ‘150-cavity’ adjacent to the active site of NA (PDB ID: 3CL0, H5N1), which was proven as an anti-influenza drug target to develop new NA inhibitors [22]. The binding modes of ten phloroglucinols with NA showed that trimeric and tetrameric phloroglucinols filixic acid ABA, ABB, and dryocrassin ABBA could dock into both the active site and the ‘150-cavity’ of NA. The other seven monomeric or dimeric phloroglucinols could only dock into the active site (Figure 2A,B). Moreover, filixic acid ABA, ABB, and dryocrassin ABBA formed several hydrogen bonds and hydrophobic interactions with the key residues Arg118, Arg152, Arg292, Arg371, Glu119, and Glu227 in the active site and ‘150-cavity’ (Figure 3A–C). Therefore, filixic acid ABA, filixic acid ABB, and dryocrassin ABBA may have the potential to inhibit NA of the H5N1 virus.

### 2.2. Isolation and Structure Characterization

**Figure 4:**
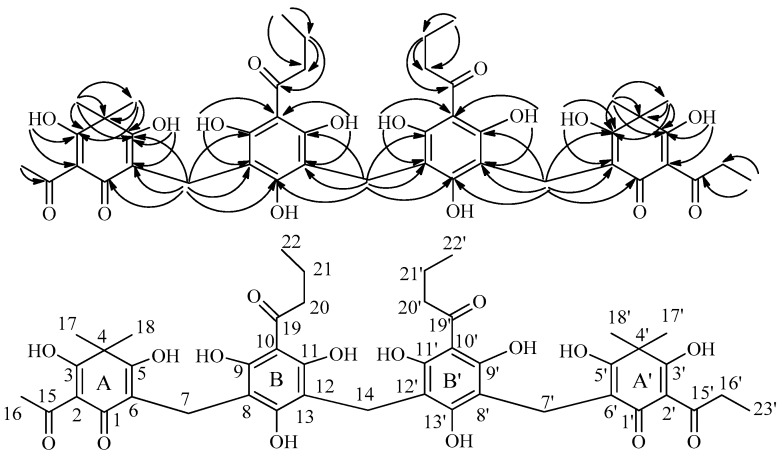
Structure of compound **7** and its HMBC correlations.

Compound **7** was obtained as a yellowish amorphous powder which showed a positive response (pink spot) to fast blue BB salt (4-benzoylamino-2,5-diethoxy-benzenediazonium salt) spray reagent on a silica gel Thin Layer chromatography (TLC) plate. The characteristic UV absorptions were at 242 and 286 nm. The absorption bands in the IR spectrum showed the presence of hydroxyl group(s) (3216 cm^−1^), carbonyl group(s) (1612 cm^−1^), phenyl group(s) (1395, 1445, 1556 cm^−1^) and alkyl group(s) (2961 cm^−1^). The quasi-molecular ion in negative high resolution electrospray ionization mass spectroscopy (HRESIMS) of **7** at *m*/*z* 833.3004 [M − H]^−^ (calcd for 833.3015) corresponded to the molecular formula of C_44_H_50_O_16_. The characteristic resonances for hydroxyl protons (δ_H_ 10.14, 11.75, 16.09, 18.54, etc.), a number of aromatic quaternary carbons (Table 2), and methylenes at δ_H/C_ 3.57/17.4 (C-7,7′)) and 3.85/17.1 (C-14) in the ^1^H- and ^13^C-NMR spectra suggested the tetrameric phloroglucinol skeleton [23,24]. ^13^C-NMR showed 25 signals, which suggested that structure of compound **7** had symmetry. The resonances at δ_H_ 3.85 (2H, H-14) are characteristic for methylenes situated between two phloroglucinol units (ring B/B′)); δ_H_ 3.57 (4H, H-7,7′)) are characteristic for methylenes situated between hexadioldienone (ring A/A′)) and phloroglucinol (ring B/B′)) units [23]. The HMBC correlations (Figure 4) of H-7,7′) (δ_H_ 3.57) with C-1,1′) (δ_C_ 188.1, 188.0), C-5,5′) (δ_C_ 172.8, 172.5), C-6,6′) (δ_H_ 111.2, 111.1), C-8,8′) (δ_C_ 105.6, 105.3), C-9,9′) (δ_C_ 160.5), and C-13,13′) (δ_C_ 158.7) confirmed the positions of methylenes between the rings A and B, and A′) and B′). The HMBC correlations of H-14 (δ_C_ 3.85) with C-12,12′) (δ_C_ 106.7), C-13,13′) (δ_C_ 158.7), and C-11,11′) (δ_C_ 159.7) confirmed the position of methylene between the rings B and B′). NMR spectral data (Table 2) of this compound resembled that of dryocrassin ABBA [25,26], suggesting they have the same skeleton.

**Scheme 1:**
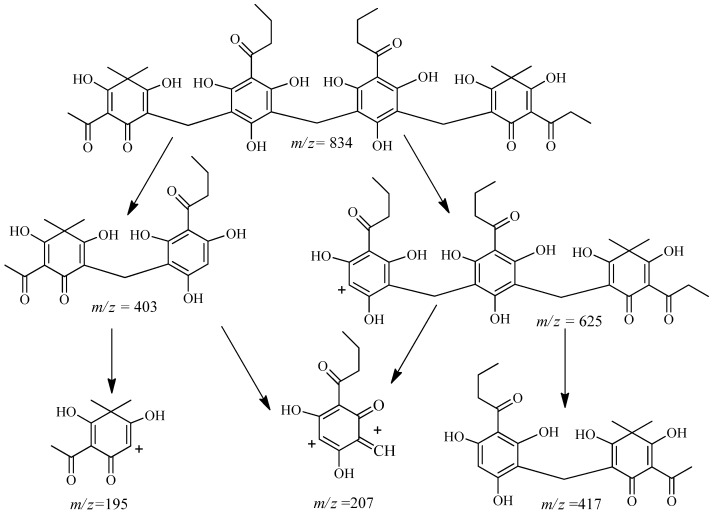
Electrospray Ionization Mass Spectroscopy (ESIMS) fragments of compound **7**.

Compared to dryocrassin ABBA, the additional resonances at δ_H_ 1.19 (H-23′)) and δ_C_ 8.4 (C-23′)) indicated that compound **7** should be dryocrassin ABBP. Although the positions of the acetyl group at C-2 in ring A, propionyl group at C-2′) in ring A′), and the two butyryl units at C-10,10′) were not supported by the HMBC spectrum, they were confirmed by the mass fragments at *m*/*z* 625, *m*/*z* 417, *m*/*z* 403, *m*/*z* 207, *m*/*z* 195 observed in the ESIMS (Scheme 1). After complete assignment of ^1^H-, ^13^C-NMR and DEPT data by the HSQC and HMBC spectra, compound **7** was elucidated as dryocrassin ABBP. It was different from the known dryocrassin ABBA, possessing a propionyl group at C-2′) instead of an acetyl group.

**Figure 5:**
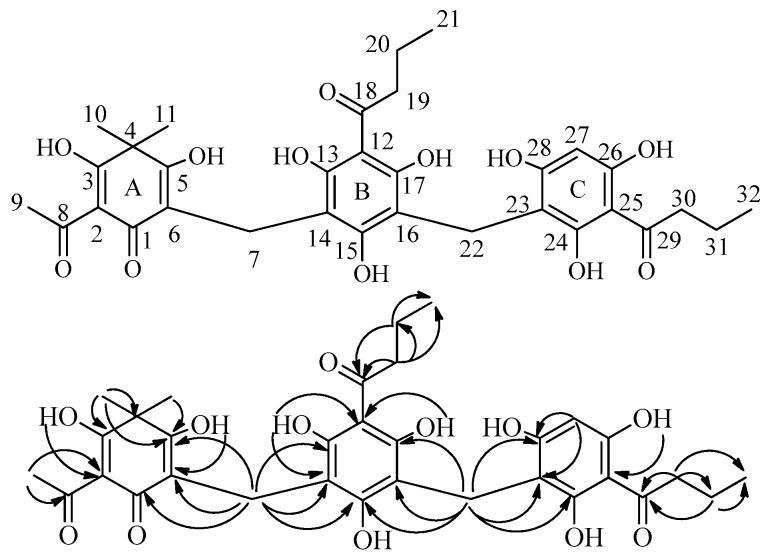
Structure of compound **13** and its HMBC correlations.

Compound **13** was obtained as an off-white powder which showed positive response (pink spot) to fast-blue BB salt spray reagent on a silica gel TLC plate. The characteristic UV absorptions were at 242 and 281 nm. The absorption bands in the IR spectrum showed the presence of hydroxyl group(s) (3243 cm^−1^), carbonyl group(s) (1611 cm^−1^), phenyl group(s) (1392, 1446 cm^−1^), and alkyl groups (2963 cm^−1^). The quasi-molecular ion in the negative HRESIMS of **13** at *m*/*z* 611.2125 [M − H]^−^ (calcd for 611.2129) corresponded to the molecular formula of C_32_H_36_O_12_. The characteristic resonances for hydroxyl protons (δ_H_ 11.65, 13.01, 16.01, 18.45, etc.), a number of aromatic quaternary carbons, and methylenes at δ_H/C_ 3.52/17.9 (C-7) and 3.80/17.1 (C-22) in the ^1^H-, ^13^C-NMR, and DEPT spectra suggested the trimeric phloroglucinol skeleton [23,24]. The resonances at δ_H_ 3.52 (2H, H-7) and δ_H_ 3.80 (2H, H-22) are characteristic for methylenes when situated between hexadioldienone (ring A) and phloroglucinol unit (ring B), and two phloroglucinol units (rings B and C), respectively [23]. The HMBC correlations (Figure 5) of H-7 (δ_H_ 3.52) with C-6 (δ_C_ 111.1), C-1 (δ_C_ 188.0), C-5 (δ_C_ 172.6), C-13 (δ_C_ 158.9), C-14 (δ_C_ 105.7), and C-15 (δ_C_ 160.5) and those of H-22 (δ_H_ 3.80) with C-15 (δ_C_ 160.5), C-16 (δ_C_ 108.5), C-17 (δ_C_ 160.7), C-24 (δ_C_ 159.6), C-23 (δ_C_ 106.7), and C-28 (δ_C_ 158.6) confirmed the sandwich positions of methylenes between the rings A, B, and C. The NMR spectral data (Table 3) of this compound resembled that of trisflavaspidic acid ABB [27], except for one methyl group absence at C-27 on ring C.

**Scheme 2:**
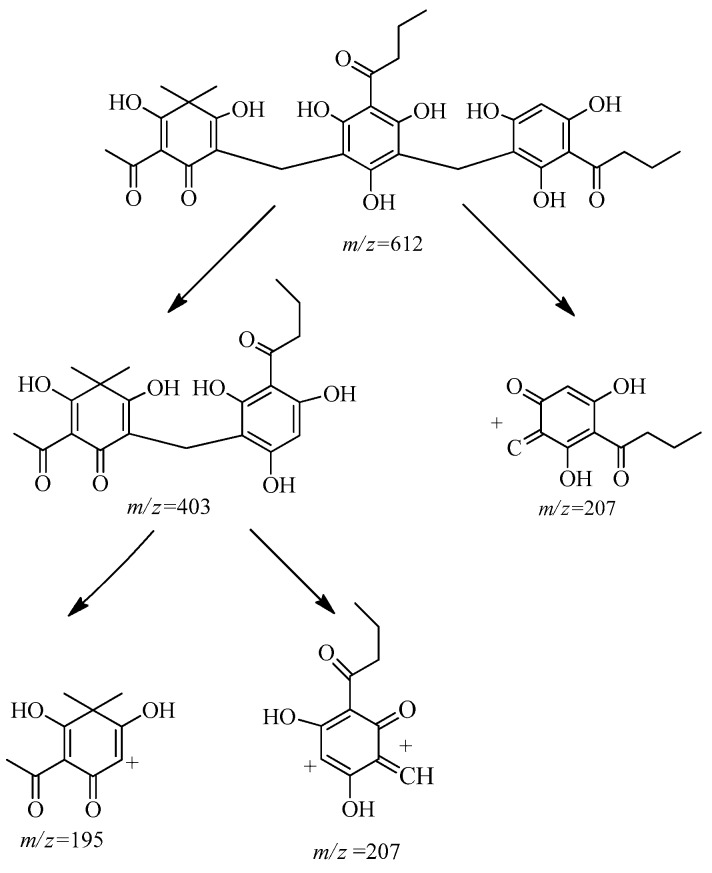
ESIMS fragments of compound **13**.

The acetyl group at C-2 was supported by the HMBC correlations of H-9 (δ_H_ 2.74) with C-2 (δ_C_ 108.2). Therefore, the two butyryl units should be located at C-12 and C-25, which were confirmed by mass fragments at *m*/*z* 403, *m*/*z* 207, and *m*/*z* 195 observed in the ESIMS (Scheme 2). After comparing the NMR resonances with those of desaspidins [28] and trisflavaspidic acid ABB, compound **13** was elucidated as nortrisflavaspidic acid ABB. It was different from the known trisflavaspidic acid ABB which did not have a methyl group (C-33) at C-27 but had a hydrogen atom on ring C.

**Figure 6:**
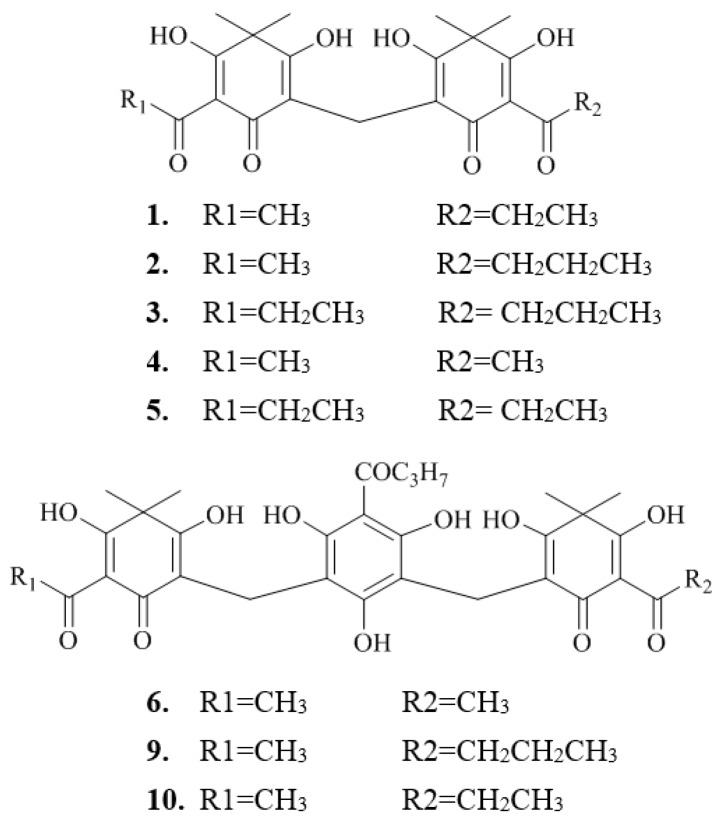

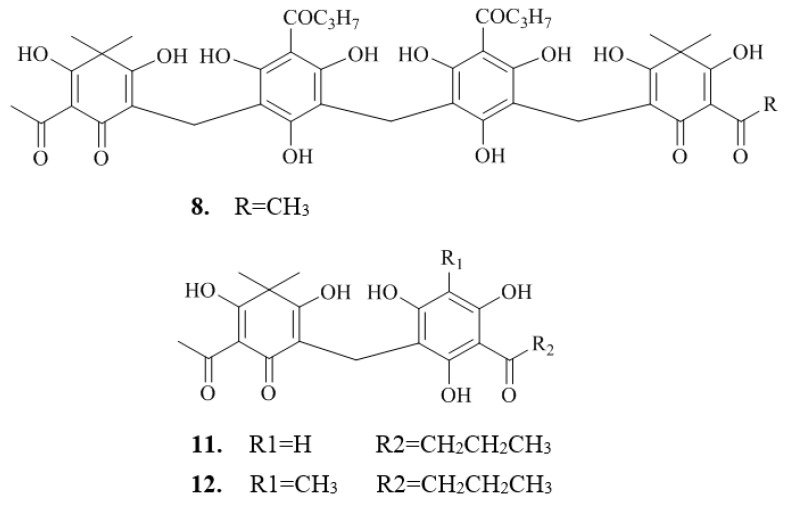
Structures of the 11 known phloroglucinols isolated from *Dryopteris crassirhizoma* (DC).

The eleven known compounds (Figure 6) were elucidated as albaspidin AP (**1**) [28,29], albaspidin AB (**2**) [30], albaspidin PB (**3**) [28,29], albaspidin AA (**4**) [31], albaspidin PP (**5**) [28,29], filixic acid ABA (**6**) [32], dryocrassin ABBA (**8**) [25,26], filixic acid ABB (**9**) [33], filixic acid ABP (**10**) [29,31], norflavaspidic acid AB (**11**) [34], and flavaspidic acid AB (**12**) [35] by using similar spectroscopic methods as described for Compounds **7** and **13**. Their spectroscopic data were compared with those reported in the literature. Compound **2** was isolated from DC for the first time.

### 2.3. Validation of Molecular Docking by NA Inhibition Assay

After virtual screening of all phloroglucinols isolated from DC via molecular docking, three of them were screened as candidates. The three candidates and two new compounds were evaluated for their NA inhibition activity, and oseltamivir was used as a positive control. The purified protein of NA (N1 Neuraminidase H274Y) from A/Anhui/1/2005 (H5N1) was used in the test. The results showed that dryocrassin ABBA and filixic acid ABA exhibited inhibitory activity against NA with IC_50_ values of 18.59 ± 4.53 and 29.57 ± 2.48 μM (Table 4), respectively. The other compounds showed moderate activity in the NA inhibition assay. However, whether the two phloroglucinols could inhibit the influenza H5N1 virus directly or not is unknown, and the molecular mechanism still needs to be clarified by further investigation.

### 2.4. Anti-Influenza Virus H5N1 Activity In Vitro

**Figure 7:**
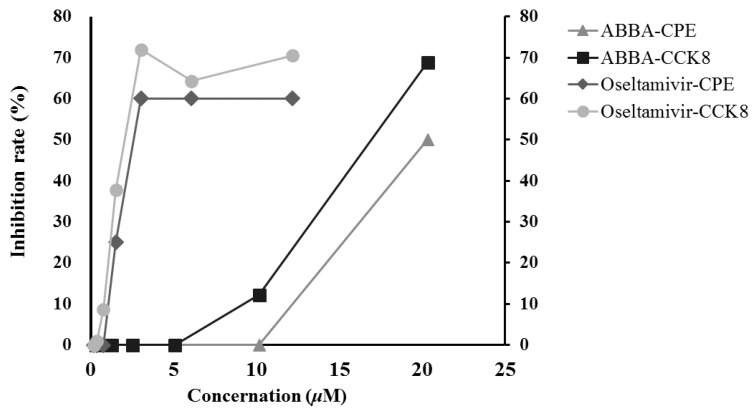
Inhibitory effect of dryocrassin ABBA on H5N1 virus in Madin-Darby canine kidney (MDCK) cells. Oseltamivir was used as a positive control. Culture medium in 96-well plates was removed and cells were washed with PBS. Then, 100 μL sample solution at different concentrations and 100 μL of virus at 100 TCID_50_ were added. After incubation at 37 °C in 5% CO_2_ for 72 h, the antiviral activity was investigated by cell counting kit-8 (CCK8) and cytopathic effect (CPE) reduction assay.

Dryocrassin ABBA and filixic acid ABA were evaluated for their antiviral activity against the H5N1(A/Vietnam/1203/2004) strain on MDCK cells via CCK8 and CPE assays in a biosafety BSL-3 laboratory, and oseltamivir was used as a positive control. The antiviral assays demonstrated that dryocrassin ABBA exhibited anti-influenza virus activity with an EC_50_ value of 16.55 μM (Figure 7), which was weaker than oseltamivir. The inhibition rate of filixic acid ABA was less than 50% at its maximal non-cytotoxic concentrations (100 μM).

### 2.5. Cellular Toxicity of Dryocrassin ABBA

**Figure 8:**
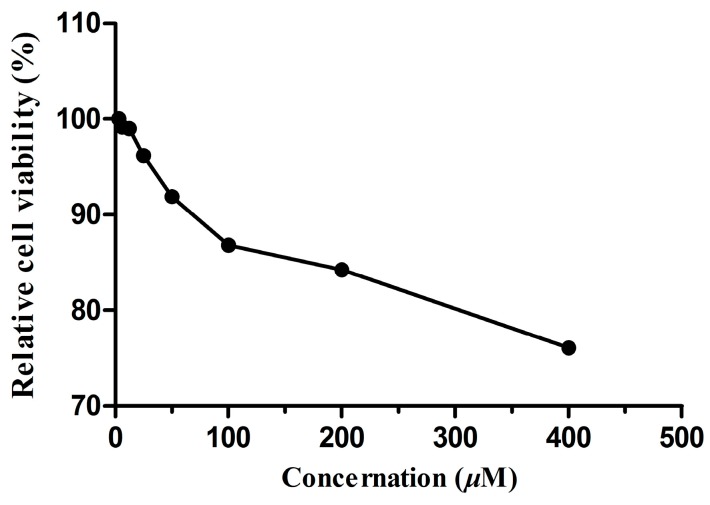
The toxicity of dryocrassin ABBA on MDCK cells. Culture medium in 96-well plates were removed and cells were washed with PBS. Then, 100 μL dryocrassin ABBA solution at different concentrations were added. After incubation at 37 °C in 5% CO_2_ for 72 h, the relative cell viability was investigated by CCK8 assay.

We evaluated the cytotoxicity of dryocrassin ABBA by CCK8 assay. MDCK cells were incubated with media in the absence or presence of dryocrassin ABBA for 72 h. The result of the cytotoxicity test showed that the 50% toxicity concentration (TC_50_) of dryocrassin ABBA was greater than 400 μM (Figure 8), which suggested that this compound might be considered as a potential therapeutic agent in the treatment of H5N1 infection, but further studies are still needed to validate its efficacy and toxicity.

## 3. Materials and Methods

### 3.1. Plant Material

Rhizome of *D. crassirhizoma* was collected in Shuangyashan (Heilongjiang province of China) in July 2014 and authenticated by Professor Chun-Sheng Liu at the School of Chinese Materia Medica, Beijing University of Chinese Medicine, where the specimen (GZDCRA02) has been deposited.

### 3.2. General Apparatus and Chemicals

Column chromatography was performed on 200–300 mesh silica gel (Qingdao Marine Chemical Co. Ltd., Qingdao, China). All purifications were monitored by TLC using commercially available glass plates pre-coated with silica gel 60 G (E. Merck Co., Billerica, Germany) and LC-6AD pHPLC (Shimadzu, Kyoto, Japan) with a UV SPD-20A detector using a reversed-phase C18 column (5 μm, 21.2 × 150 mm; Phenomenex Inc., Torrance, CA, USA). All the silica gel and GF_254_ plates were treated with citrate buffer (pH 2.0). The analytical HPLC was performed using an Agilent LC1100 (Agilent Technologies, Santa Clara, CA, USA) equipped with a MWD detector using a reversed-phase C18 column (5 μm, 4.6 × 250 mm; Phenomenex Inc., Torrance, CA, USA). ^1^H- and ^13^C-NMR data were all obtained on an Advance DRX-500 spectrometer (Bruker, Rheinstetten, Germany) in CDCl_3_ with TMS as an internal standard. HRESIMS was performed on a QTOF-MS (AB SCIEX, Framingham, MA, USA). Anti-influenza virus H5N1 activity was assayed with a SpectraMax M3 microplate reader (Molecular Devices, Sunnyvale, CAUSA).

### 3.3. Viruses, Cells and Reagents

The purified protein of NA (N1 Neuraminidase H274Y) from A/Anhui/1/2005 (H5N1) was purchased from Sino Biological Inc. (Beijing, China). Influenza virus strain A/Vietnam/1203/2004 (10^8.5^TCID_50_/mL, H5N1) and Madin-Darby canine kidney (MDCK) cells were provided by the Institute of Laboratory Animal Science, CAMS&PUMC (Beijing, China). The experiments were performed at a BSL-3 laboratory in the Institute of Laboratory Animal Science, Chinese Academy of Medical Sciences & Peking Union Medical College. Viruses were propagated on MDCK cells and incubated in Dulbecco’s Modified Eagle Media (DMEM) supplemented with 10% fetal bovine serum (FBS) and 0.01% antibiotic-antimycotic solution (100 U/mL penicillin, and 100 μg/mL streptomycin) at 37 °C. Antibiotic-antimycotic solution, trypsin-EDTA, FBS, and DMEM were supplied by GIBCO (USA). Oseltamivir phosphate capsules were obtained from Roche Pharmaceuticals Ltd. (Shanghai, China). The stock solution of oseltamivir was prepared as follows: The capsule content powder (50 mg) was dissolved in 50 mL distilled water by ultrasonication and was centrifuged. 4 mL 20% NaOH was added to 32 mL of the supernatant and stirred at room temperature for 6 h. Then, the pH value of the solution was adjusted to 6.5 with glacial acetic acid [36]. Cell Counting Kit-8 (CCK8) was purchased from Dojindo (Japan). MES and 2′-(4-Methylumbelliferyl)-α-*N*-acetylneuraminic (4-MUNANA) were purchased from Sigma (USA). Stock solutions of the compounds were made in dimethyl sulfoxide (DMSO) and subsequently diluted in DMEM. The final DMSO concentration was a maximum of 0.1%, which had no effect on the cell cultures, and 0.1% DMSO was also added to all the no-drug control samples.

### 3.4. Molecular Docking

Molecular docking is a well-recognized method for finding the optimal conformation and orientation of a ligand when binding to a receptor and has been extensively validated over the years [37,38,39]. In this research, AutoDock 4.2 and AutoDock Vina were used to evaluate the binding affinity of the phloroglucinols isolated from DC with NA (PDB ID 3CL0) of influenza virus H5N1.

AutoDock Tools-1.5.6 was used to prepare the receptor by removing the bound ligand (oseltamivir) and crystallographic water molecules from the active site of the protein, and by adding all polar hydrogen atoms and the charges to the macromolecule. Gasteiger charges were calculated for each atom of the receptor molecule in AutoDock 4.2. The grid map was centered at the active site pocket of the protein by running the Auto Grid file. We used grid maps with a grid box size of 72 × 92 × 64 with a grid-point spacing of 0.375 Å. During docking, center grid parameters were specified for the *x*, *y*, and *z* axis as −31.409, −56.287, and 8.115, respectively. Docking calculations were performed by Autodock Vina. The default settings for docking in Vina were used except for num_modes (20) and exhaustiveness (100). The empirical scoring function and the iterated local search for global optimization of Vina were employed here to achieve a significantly improved calculation speed and better efficiency compared with AutoDock 4.2 [40]. After extracting the binding ligand oseltamivir, the X-ray structure of NA was used for redocking with oseltamivir and the score was calculated to check the accuracy of the AutoDock Vina program. Twenty-three phloroglucinols were prepared to dock into the active site of NA. The highest scored conformation was selected as the potential bioactive conformation. Additionally, the schematic plan of ligand interactions showing hydrogen bonds and hydrophobic interactions were visualized by LigPlot^+^ and PyMOL 1.5.0.4.

### 3.5. Extraction and Isolation

The dried rhizome (15 kg) of DC was refluxed with 90% EtOH three times (2, 2, and 1 h, respectively). The combined extracts were evaporated under reduced pressure at 35 °C to yield a dark brown syrupy residue (2.92 kg), which was suspended in H_2_O (3 L), and then filtered. The filtrate was sequentially partitioned with petroleum ether (Pet), chloroform, and EtOAc, respectively, to yield 436 g Pet extract, 119 g chloroform extract, 111 g EtOAc extract, 1046 g water extract, and 202 g precipitate. The precipitation fraction was subjected to column chromatography (CC) on a silica gel column (54.0 cm × 9.4 cm, 200–300 mesh), and gradient eluted with *n*-hexane/chloroform (8:1–1:1) to give sixteen fractions (A1–P1). The fraction K1 was divided into seven parts (A2–G2) by a silica gel column (54.0 cm × 5.0 cm, 200–300 mesh), and gradient eluted with *n-*hexane/chloroform (3:1–1:1). A portion of D2 (0.87 g) was subjected to a silica gel column (45.0 cm × 1.2 cm, 200–300 mesh) with *n*-hexane/chloroform (16:1) to obtain 3 sub-fractions (d1, d2, d3). Fraction d1 was purified by preparative High Performance Liquid Chromatography (pHPLC) using MeOH/0.1%TFA (94:6) as the eluent to afford compound **1** (51 mg), compound **2** (5.3 mg), and compound **3** (3.1 mg). Fraction d2 was applied to pTLC [silica gel 60 G (4.5 g/plate), glass plates (15.0 cm × 15.0 cm)], developed with *n-*hexane/chloroform (1:4), and visualized under UV at 365 nm, then further purified by pHPLC with MeOH/0.1%TFA (93:7) to afford compound **4** (25.1 mg). Compound **5** (10.7 mg) was obtained from part d3 by pTLC, developed with *n*-hexane/acetone (25:1) and visualized under UV at 365 nm, and purified by pHPLC with MeOH/0.1% TFA (94:6). A portion of G2 was separated by pTLC, developed with n-hexane/acetone (15:1), and further purified by pHPLC with MeOH/0.1% TFA (99:1) to afford compound **6** (96.6 mg). Compound **7** (18 mg), compound **8** (896 mg), and compound **9** (15.6 mg) were obtained from the precipitate by pTLC, developed with *n*-hexane/chloroform/methanol (30:15:1) and n-hexane/acetone (20:1), and visualized under UV at 365 nm.

The chloroform fraction (119 g) was subjected to a silica gel column (72.5 cm × 10 cm, 200–300 mesh), gradient eluted with n-hexane/acetone (32:1–1:1) to give seven fractions, GZ-1–GZ-7. The fraction GZ-2 (2.72 g) was separated by pTLC, developed with *n*-hexane/acetone (64:1) and recrystallized to give compound **10** (17 mg). A portion of GZ-4 (6.0 g) was subjected to a medium voltage silica gel column (46.0 cm × 4.9 cm, 200–300 mesh), and gradient eluted with Pet/acetone (50:1-1:1) to obtain 4 sub-fractions (GZ-4-1–GZ-4-4). The GZ-4-2 was separated by pHPLC with MeOH/0.1% TFA (95:5) to give compound **11** (25.3 mg). Compound **12** (13.4 mg) and compound **13** (15.3 mg) were obtained from GZ-5 (26.0 g) by a medium voltage silica gel column (46.0 cm × 7.0 cm, 200–300 mesh), gradient eluted with n-hexane/acetone/ethylacetate (60:1:1–3:1:1) and prepared with pTLC (Pet/ethylacetate = 3:1; *n*-hexane/acetone = 1:1).

Compound (**7**): yellowish amorphous powder, UV (CHCl_3_) λ_max_: 242, 286 nm; IR (FT-IR) *ν*_max_ 3216, 2961, 1612, 1556, 1445, 1395, 1323, 1289, 1194, 1161, 898, 610 cm^−1^; for ^1^H- and ^13^C-NMR data in CDCl_3_, see Table 2; HRESIMS *m*/*z* 833.3004 [M − H]^−^ (Calcd. for C_44_H_49_O_16_, 833.3015).

Compound (**13**): off-white powder, UV (CHCl_3_) *λ*max 242, 281 nm; IR (FT-IR) ν_max_ 3243, 2963, 1611, 1446, 1392, 1223, 1150, 896, 822, 609 cm^−1^; for ^1^H- and ^13^C-NMR data in CDCl_3_, see Table 3; HRESIMS *m*/*z* 611.2125 [M − H]^−^ (Calcd. for C_32_H_35_O_12,_ 611.2129).

### 3.6. NA Inhibition Assay

The NA inhibition assay was conducted using purified protein NA (1 U/mL) from the influenza virus strain A/Anhui/1/2005 H5N1. The inhibition activity of the three candidates and two new compounds on NA was assayed by quantifying the fluorescent product resulting from the cleavage of the substrate 4-MUNANA. The reported method was adopted with some modifications [41]. The reaction mixture consisted of the tested phlorolucinols, NA and 4-MUNANA in 33.3 mM MES buffer (containing 4 mM CaCl_2_, pH 6.5) in a 96-well plate. After incubation for 30 min at 37 °C, the reaction was terminated with 100 μL 0.15 M glycine buffer (pH 10.3) and the fluorescence of the mixture was recorded for the excitation wavelength of 355 nm and emission wavelength of 460 nm [42]. The inhibition ratio was obtained using the equation: Inhibition activity (%) = (*F*_NA_ − *F*_Sample_)/(*F*_NA_ − *F*_Substrate_) × 100%, where *F*_NA_ is the fluorescence of the influenza virus NA control (NA, buffer, and substrate), *F*_Substrate_ is the fluorescence of the substrate control (buffer and substrate), and *F*_Sample_ is the fluorescence of the tested samples (NA, sample solution, and substrate). Subsequently, the 50% inhibitory concentration (IC_50_) was determined by extrapolation of the results from various doses tested using a linear equation. The experiment was repeated three times with a similar finding each time. The three independent measurements were collected to determine the mean and SD values.

### 3.7. Anti-Influenza Virus H5N1 Activity in Vitro

Assays of antiviral activity were performed using the CCK8 and CPE methods. Oseltamivir was used as a positive control. The CCK8 and CPE assays were performed in a biosafety BSL-3 laboratory as follows. MDCK cells were grown in a 96-well culture plate (10^4^ cells/well) for 24 h. Then, the medium was removed and washed with PBS, and 100 μL sample solution at different concentrations and 100 μL virus at 100 TCID_50_ were inoculated onto MDCK cells for 72 h at 37 °C under 5% CO_2_. After 3 days, we observed the cytopathic effect by a Fluorescence Inversion Microscope System and recorded the results, and then the solution was removed and the cells were washed with PBS, and then 190 µL DMEM and 10 μL CCK8 were added to each well. The plates were incubated in the dark for 3 h at 37 °C. The absorbance was read at 450/630 nm using a microplate reader. All tests were performed in quadruplicate. The inhibition ratio was determined as follows: inhibition activity (%) = (OD_sample_ – OD_virus_)/(OD_cellular control_ − OD_virus_) × 100%, where OD_sample_ is the optical density of the tested sample at a certain concentration, OD_virus_ is the optical density of the influenza virus control, and OD_cellular control_ is the optical density of normal cells. The 50% inhibition concentration (IC_50_) was determined by linear extrapolation of the results from various doses tested.

### 3.8. Cytotoxicity Assay

The CCK8 method was used to measure the cytotoxicity of the tested sample. MDCK cells were grown in a 96-well plate 200 µL (10^4^ cells/well) for 24 h. The culture supernatant was replaced with maintenance medium containing the tested samples at various concentrations. After 72 h incubation, we removed the culture supernatant and added 190 μL DMEM and 10 μL CCK8 to each well. The plates were incubated at 37 °C for 3 h. The fluorescence intensity was recorded with a microplate reader at 450/630 nm. All tests were performed in quadruplicate. The 50% toxic concentration (TC_50_) was calculated by regression analysis.

## 4. Conclusions

This is the first comprehensive investigation of anti-influenza virus H5N1 activity on all phloroglucinols from the rhizome of *D. crassirhizoma* by molecular docking. Experimental results indicate that dryocrassin ABBA may have the potential to be used against influenza virus H5N1 infection which will have to be verified in further detailed studies. Two new phloroglucinols were obtained and they also have moderate inhibitory activity on NA.

## Figures and Tables

**Table 1:** Vina energy scores of the screened ten phloroglucinols bound to NA.

Compound	MF	MW	Affinity (Kcal/mol)
Dryofragin	C_28_H_38_O_5_	454	−8.0
Albaspidin AA	C_21_H_24_O_8_	404	−8.0
Albaspidin AB	C_23_H_28_O_8_	432	−8.1
Albaspidin AP	C_22_H_26_O_8_	418	−8.0
Flavaspidic acid AB	C_22_H_26_O_8_	418	−8.2
Flavaspidic acid PB	C_23_H_28_O_8_	432	−8.1
Norflavaspidic acid AB	C_21_H_24_O_8_	404	−8.0
Filixic acid ABA	C_32_H_36_O_12_	612	−8.0
Filixic acid ABB	C_32_H_36_O_12_	640	−8.1
Dryocrassin ABBA	C_43_H_48_O_16_	820	−8.3

**Table 2:** ^1^H- and ^13^C-NMR spectroscopic data for compounds **7** and **8** in CDCl_3_.

Position	Compound 7		Compound 8	
	δ_H_	δ_C_	δ_H_	δ_C_
1 (1′)		188.1 (188.0)		188.0
2 (2′)		108.2 (108.1)		108.2
3 (3′)	18.54 (OH)	199.2 (198.5)	18.52 (OH)	199.3
4 (4′)		44.5 (44.3)		44.5
5 (5′)	9.71 (OH)	172.8 (172.5)	9.80 (OH)	172.8
6 (6′)		111.2 (111.1)		111.2
7 (7′)	3.57 s	17.4	3.57 s	17.4
8 (8′)		105.6 (105.3)		105.7
9 (9′)	16.09 (OH)	160.5	16.10 (OH)	160.6
10 (10′)		158.9		158.9
11 (11′)	11.75 (OH)	159.7	11.73 (OH)	159.7
12 (12′)		106.7		106.7
13 (13′)	10.14 (OH)	158.7	10.12 (OH)	158.7
14	3.85 s	17.1	3.87 s	17.1
15		203.6		203.5
15′		207.5		203.5
16	2.75 s	29.7	2.77 s	29.4
16′	3.21 m	34.9	2.77 s	29.4
17 (17′)	1.43 s	25.3	1.46 s	25.3
18 (18′)	1.53 s	24.1	1.57s	24.1
19 (19′)		208.2 (208.1)		208.3
20 (20′)	3.21 m	46.0	3.22 m	46.1
21 (21′)	1.73 m	18.0 (17.8)	1.76 m	17.9
22 (22′)	1.01 t	14.0	1.03 t	14.0
23′	1.19 t	8.4		

**Table 3:** ^1^H- and ^13^C-NMR spectroscopic data for compounds **13** and trisflavaspidic acid ABB in CDCl_3_.

Position	Compound 13		Trisflavaspidic Acid ABB	
	δ_H_	δ_C_	δ_H_	δ_C_
1		188.0		188.2
2		108.2		108.5
3	18.45 (OH)	199.1	18.34 (OH)	199.3
4		44.4		44.7
5	18.46 (OH)	172.6	18.34 (OH)	172.7
6		111.1		111.4
7	3.52 s	17.9	3.46 s	17.4
8		203.5		203.7
9	2.74 s	29.7	2.68 s	29.6
10	1.43 s	25.2	1.37 s	25.5
11	1.55 s	24.1	1.47 s	24.4
12		106.8		106.4
13	16.01 (OH)	159.6	16.01 (OH)	159.8
14		106.7		107.0
15	9.55 s	160.5	9.60 s	160.7
16		108.5		108.6
17	16.00 (OH)	160.7	15.97 (OH)	160.2
18		208.0		208.2
19	3.05 t	46.0	3.08 t	46.2
20	1.73 m	18.0	1.66 m	18.4
21	1.01 m	14.0	0.94 m	14.2
22	3.80 s	17.1	3.76 s	17.0
23		105.7		105.9
24	13.01 s	158.9	12.94 s	159.0
25		105.3		105.6
26	11.65 s	159.5	11.59 s	159.1
27	5.90 s	96.6		102.0
28	10.09 (OH)	158.6	10.05 (OH)	158.9
29		206.7		207.1
30	3.15 t	45.6	3.08 t	46.0
31	1.73 m	18.0	1.66 m	18.1
32	1.01 m	14.0	0.94 m	14.1
33			2.03	7.7

**Table 4:** Inhibitory effects of the five phloroglucinols on influenza virus (H5N1) NA.

Compounds	H5N1 (A/Anhui/1/2005)
	IC_50_ (μM)
Dryocrassin ABBA	18.59 ± 4.53
Filixic acid ABA	29.57 ± 2.48
Filixic acid ABB	96.58 ± 7.67
Dryocrassin ABBP	48.90 ± 2.91
Nortrisflavaspidic acid ABB	51.49 ± 6.84
Oseltamivir (nM)	4.62 ± 0.77

## Supplementary Materials

Structures of the twenty-three phloroglucinols, Table S1; UV, IR, ^1^H-NMR, ^13^C-NMR, DEPT, HSQC, HMBC, and HRESIMS spectra are available as supporting data, Figures S1–S16. Supplementary materials are available online.
