# Supplementary material for: Anti-Influenza Virus (H5N1) Activity Screening on the Phloroglucinols from Rhizomes of Dryopteris crassirhizoma
Source: Molecules. 2017 Mar 8;22(3):431. doi: 10.3390/molecules22030431 (PMC6155227; doi:10.3390/molecules22030431)
Supplement: Supplementary file 1 [file molecules-22-00431-s001.pdf]

Supplementary Materials: Anti-influenza Virus (H5N1) Activity Screening on the Phloroglucinols from Rhizomes of *Dryopteris crassirhizoma*

Juan Wang, Yan-Tao Yan, Shen-Zhen Fu, Bing Peng, Lin-Lin Bao,

Yan-Ling Zhang, Jing-Hong Hu, Zu-Ping Zeng, Dong-Hao Geng, Zeng-Ping Gao

Table S1, Figure S1-S16 are as below.

Table S1 Structures of twenty-three phloroglucinols

NO.	Compounds	Structure
1	Picraquassioside D	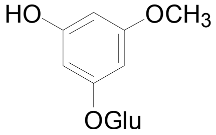
2	Dryopteroside	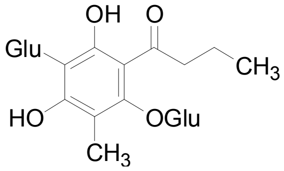
3	3-Methyl-butyrylphloroglucinol	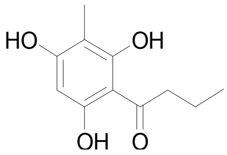
4	Aspidinol-B	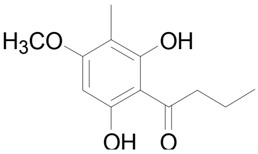
5	Dryofragin	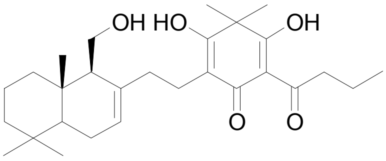

6	Phloropyron BB	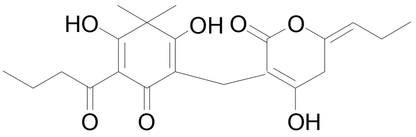
7	albaspidin AA	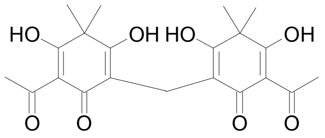
8	albaspidin AB	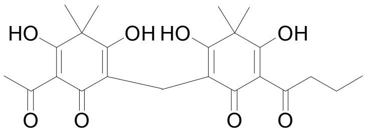
9	albaspidin AP	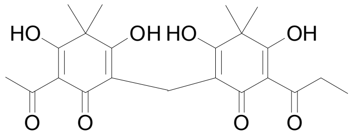
10	albaspidin PB	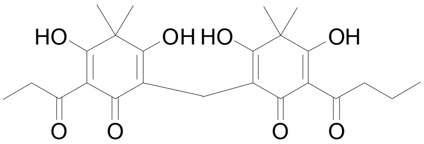
11	albaspidin PP	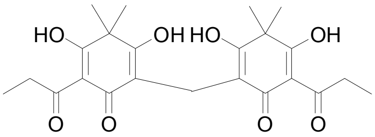
12	flavaspidic acid AB	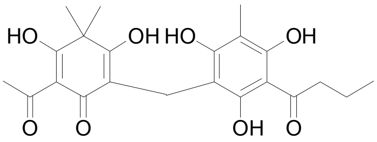
13	Norflavaspidic acid AB	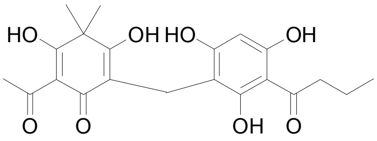
14	Flavaspidic acid PB	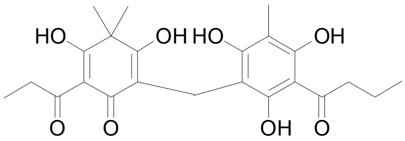
15	aspidin BB	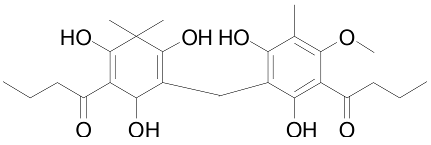

16	Aemulin BB	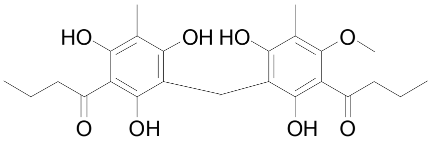
17	Methylene-bis-methylphlorobutyrophenone	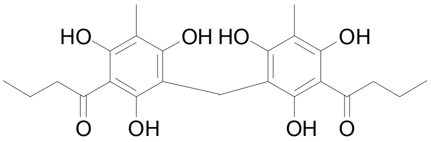
18	filixic acid ABA	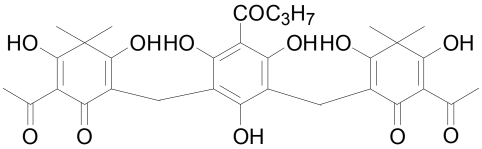
19	filixic acid ABP	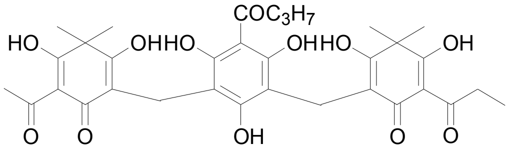
20	filixic acid ABB	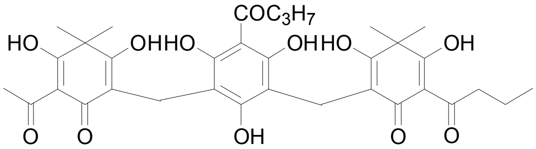
21	Trisflavaspidic acid ABB)	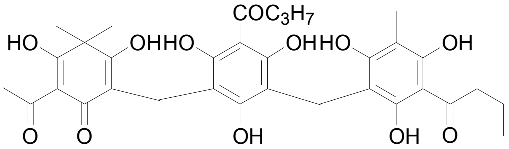
22	Dryocrassin ABBA	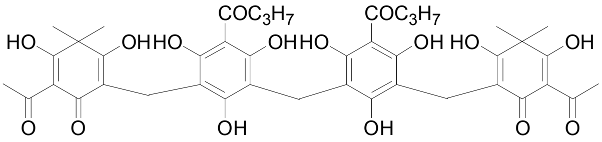
23	Penta-albaspidin ABBBA	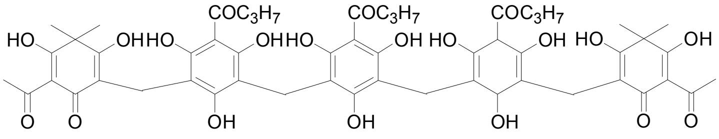

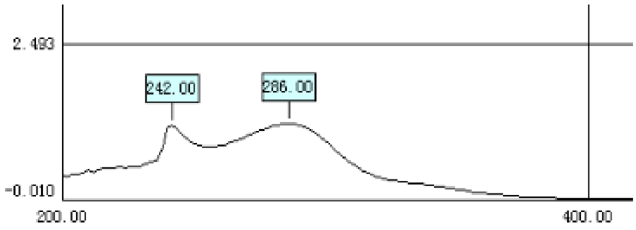

Figure S1. UV absorption spectrum of compound **7**

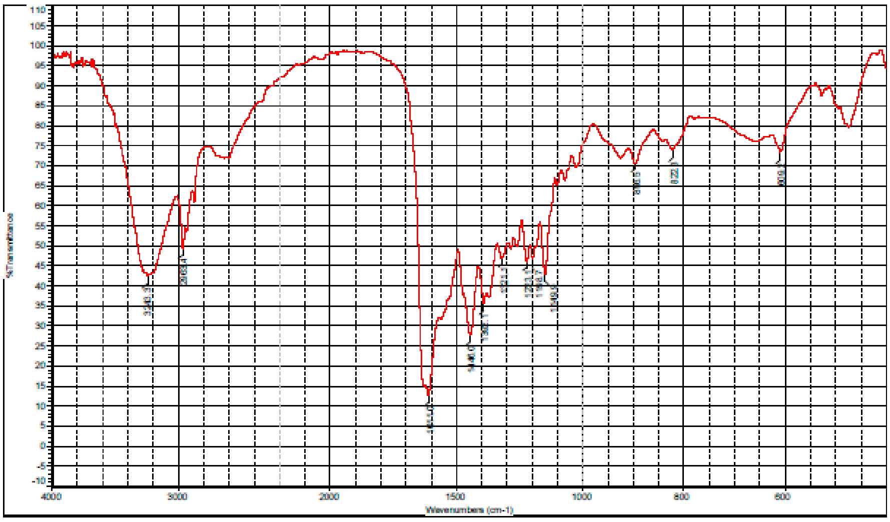

Figure S2. IR absorption spectrum of compound **7**

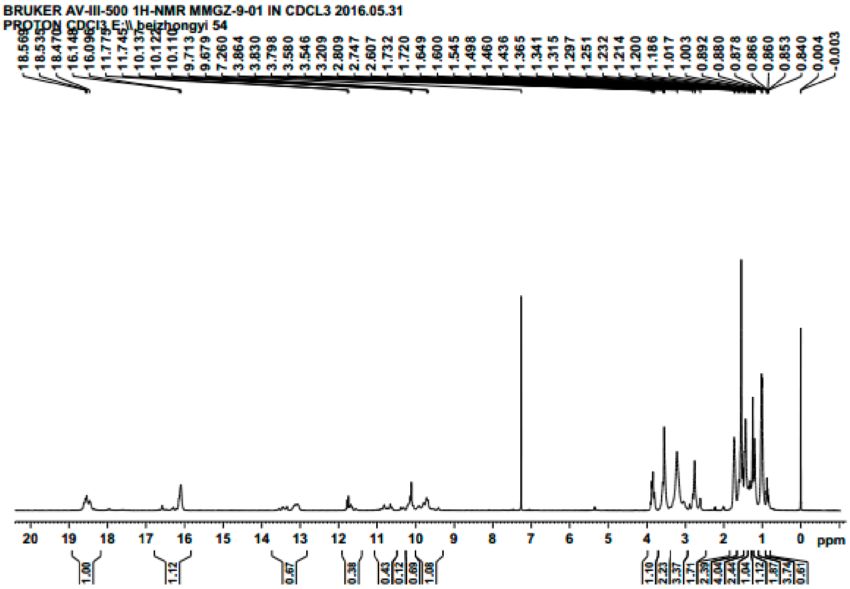

Figure S3. The ^{1}H NMR spectrum of compound **7**

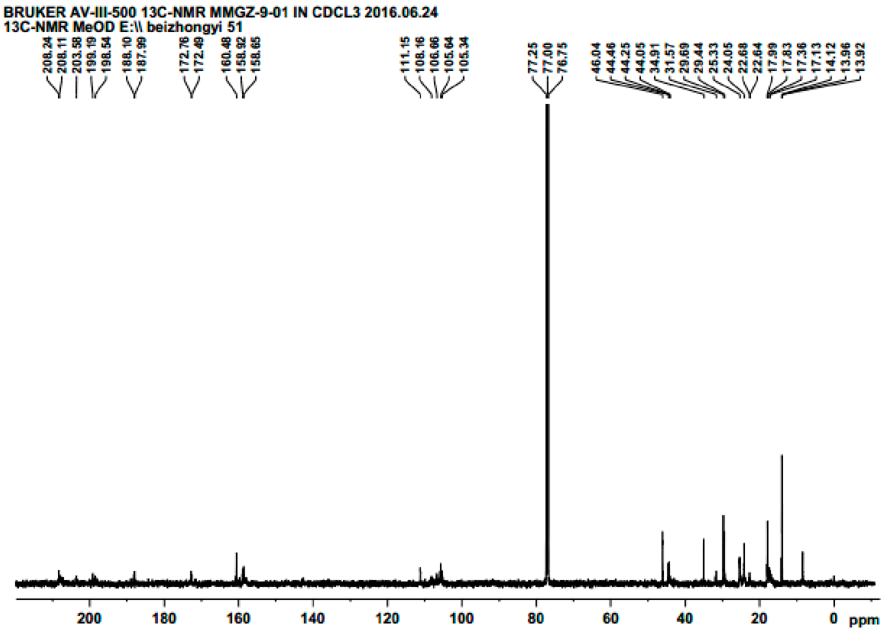

Figure S4. The ^{13}C NMR spectrum of compound **7**

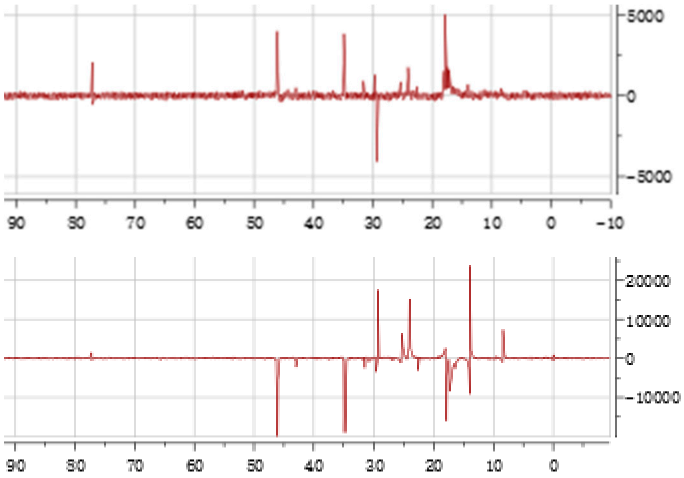

Figure S5. The DEPT spectrum of compound **7**

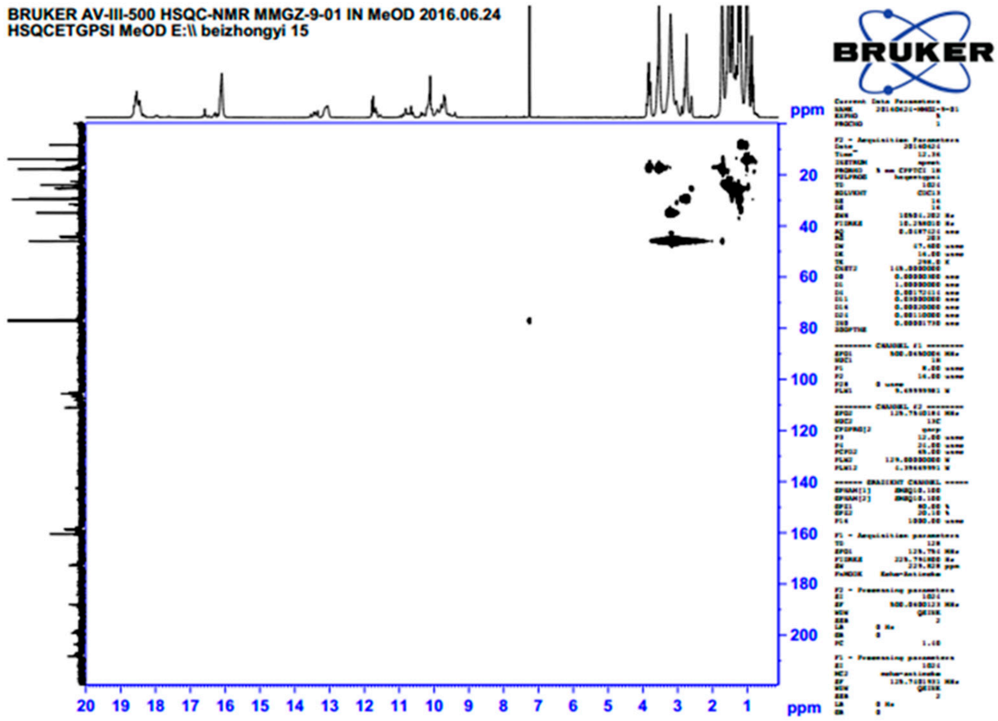

Figure S6. The HSQC spectrum of compound **7**

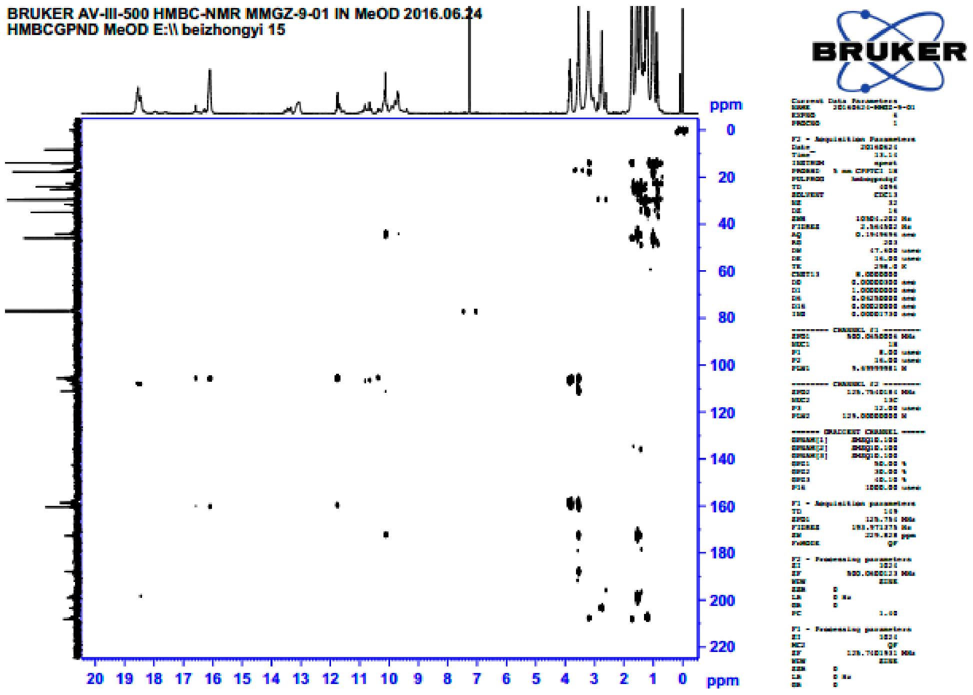

Figure S7. The HMBC spectrum of compound **7**

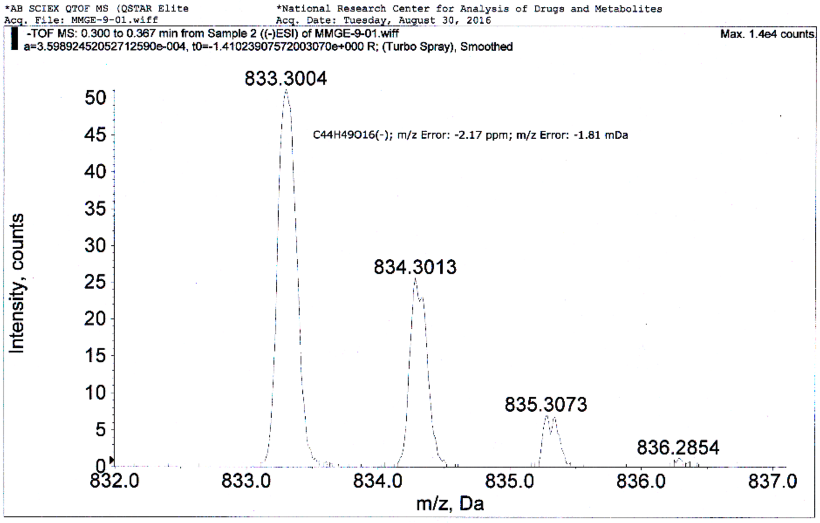

Figure S8(A). The HR-ESI-MS spectrum of compound **7**

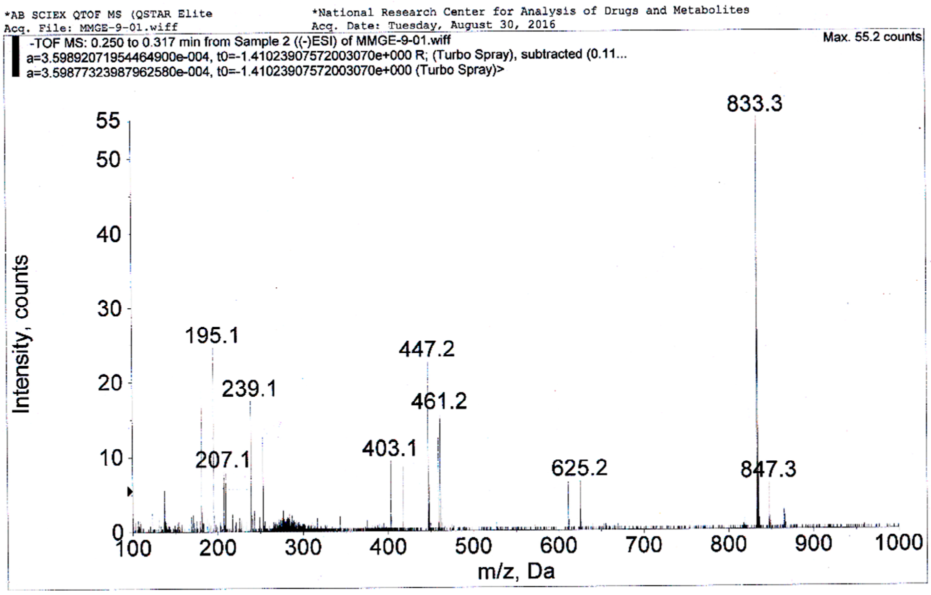

Figure S8(B). The ESI-MS fragments of compound **7**

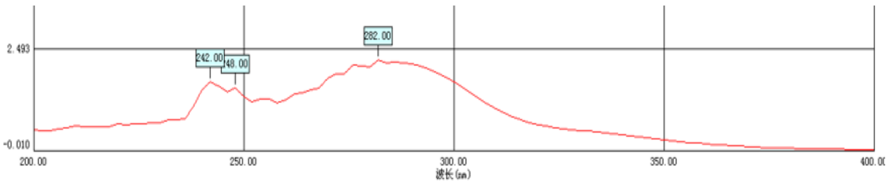

Figure S9. The UV spectrum of compound **13**

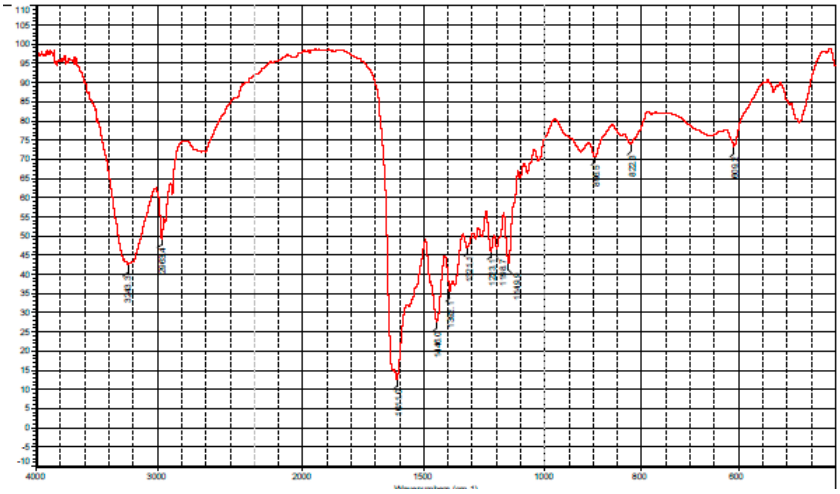

Figure S10. The IR spectrum of compound **13**

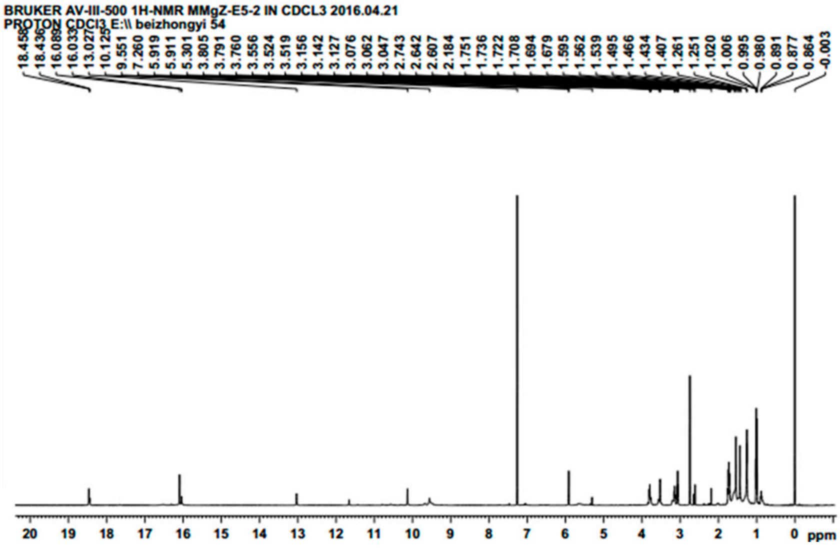

Figure S11. The ^{1}H NMR spectrum of compound **13**

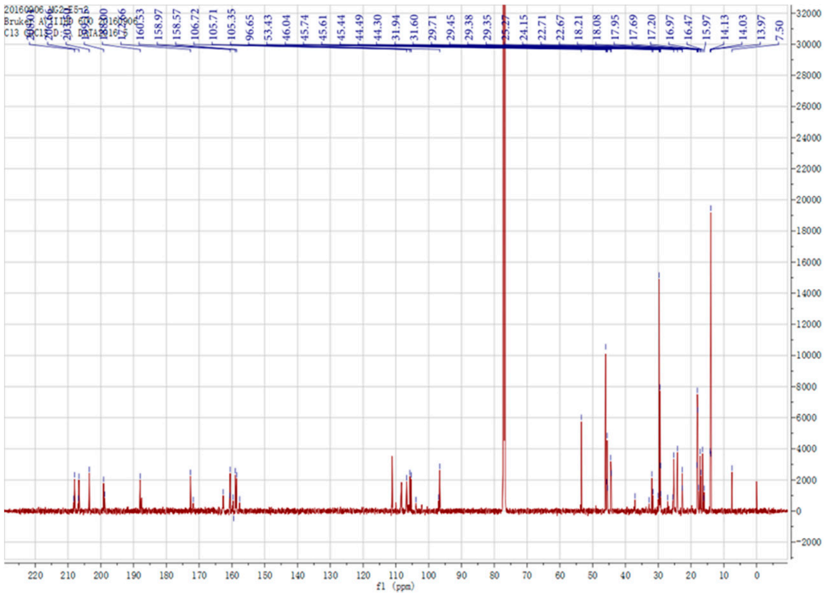

Figure S12. The ^{13}C NMR spectrum of compound **13**

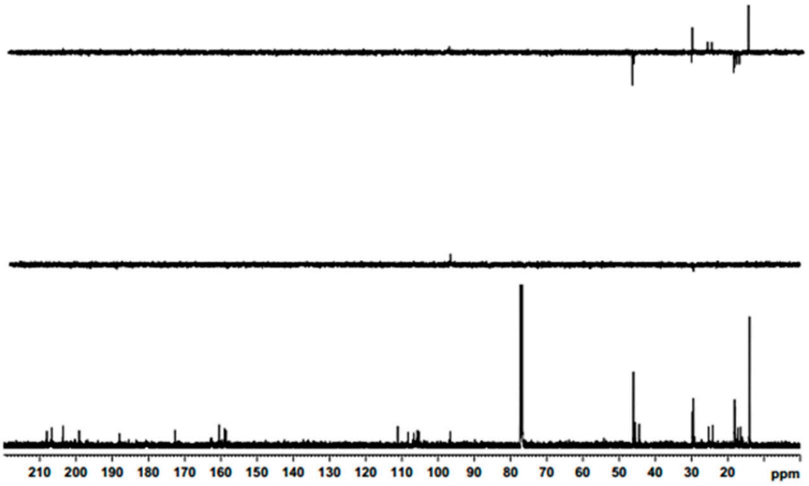

Figure S13. The DEPT spectrum of compound **13**

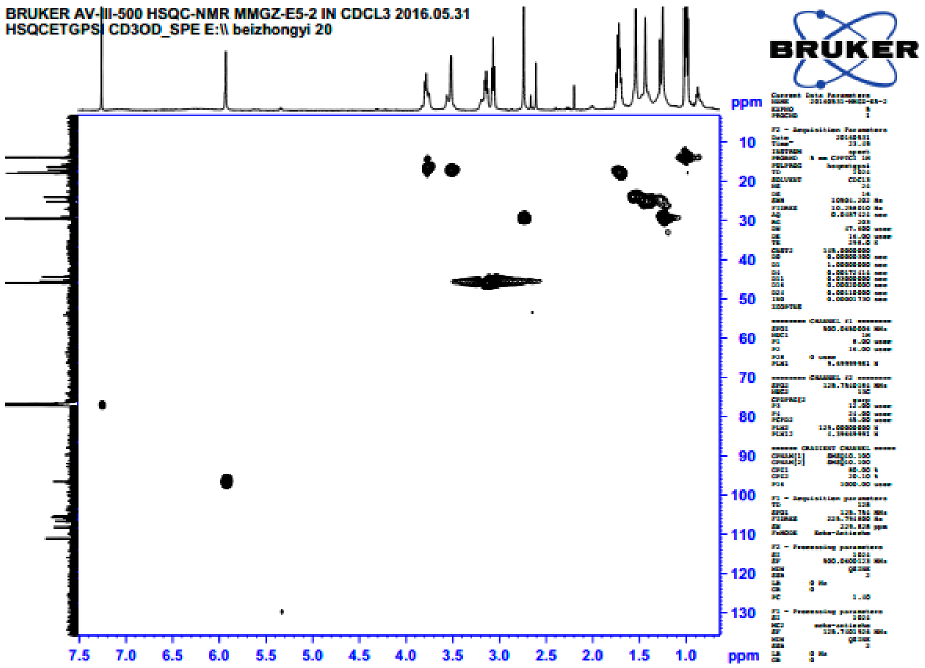

Figure S14. The HSQC spectrum of compound **13**

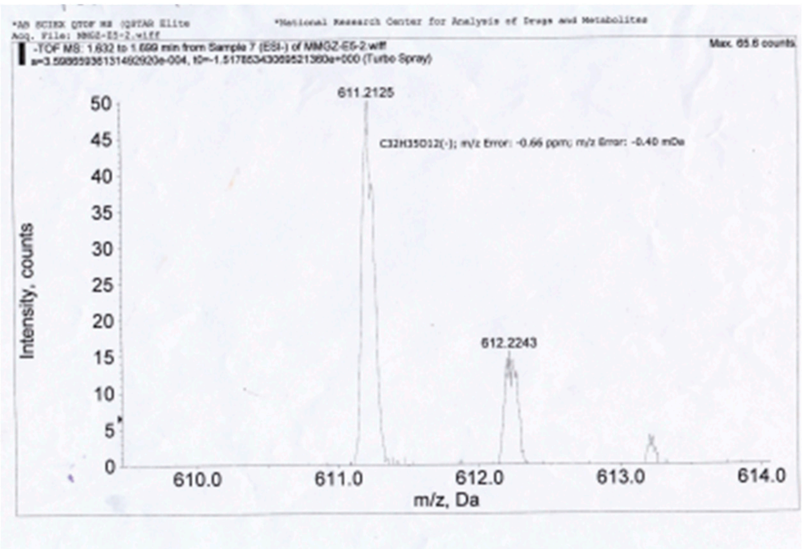

Figure S15. The HMBC spectrum of compound **13**

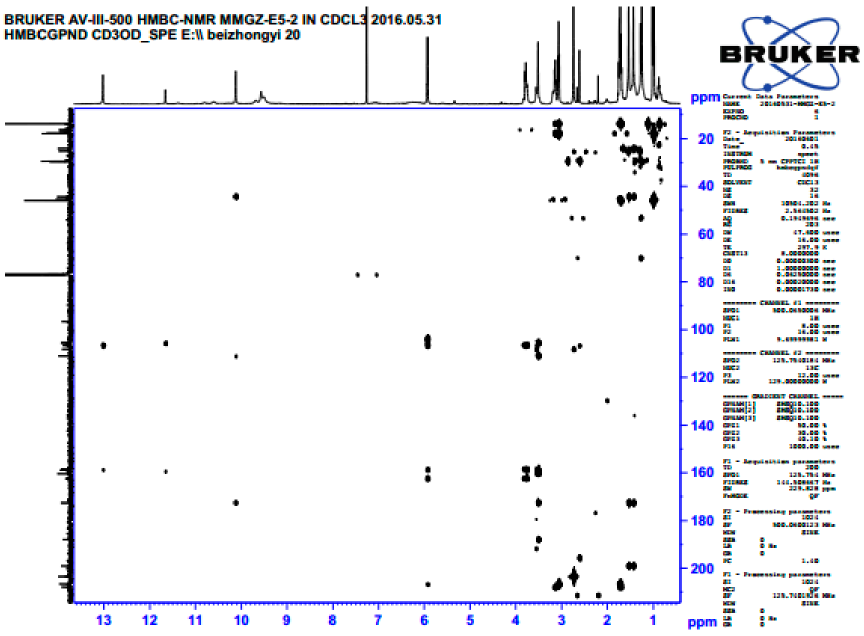

Figure S16(A). The HR-ESI-MS of compound **13**

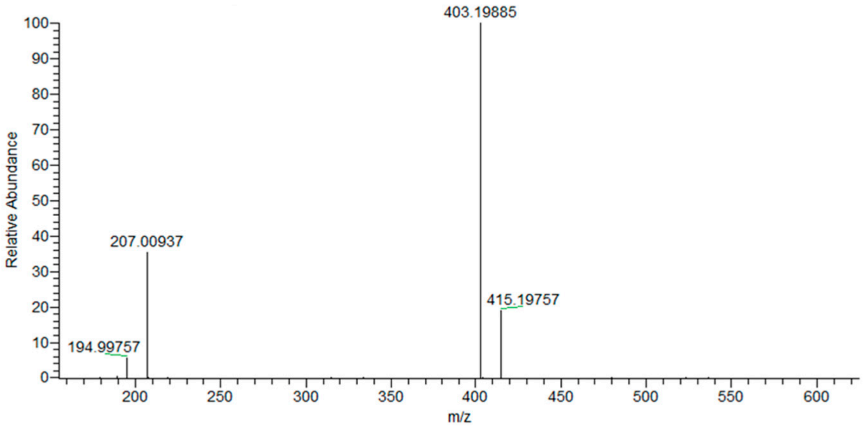

Figure S16(B). The HR-ESI-MS fragments of compound **13**
